# iLOCi: a SNP interaction prioritization technique for detecting epistasis in genome-wide association studies

**DOI:** 10.1186/1471-2164-13-S7-S2

**Published:** 2012-12-07

**Authors:** Jittima Piriyapongsa, Chumpol Ngamphiw, Apichart Intarapanich, Supasak Kulawonganunchai, Anunchai Assawamakin, Chaiwat Bootchai, Philip J Shaw, Sissades Tongsima

**Affiliations:** 1 National Center for Genetic Engineering and Biotechnology, Pathumthani, 12120, Thailand; 2 National Electronics and Computer Technology Center, Pathumthani, 12120, Thailand

## Abstract

**Background:**

Genome-wide association studies (GWAS) do not provide a full account of the heritability of genetic diseases since gene-gene interactions, also known as epistasis are not considered in single locus GWAS. To address this problem, a considerable number of methods have been developed for identifying disease-associated gene-gene interactions. However, these methods typically fail to identify interacting markers explaining more of the disease heritability over single locus GWAS, since many of the interactions significant for disease are obscured by uninformative marker interactions e.g., linkage disequilibrium (LD).

**Results:**

In this study, we present a novel SNP interaction prioritization algorithm, named iLOCi (Interacting Loci). This algorithm accounts for marker dependencies separately in case and control groups. Disease-associated interactions are then prioritized according to a novel ranking score calculated from the difference in marker dependencies for every possible pair between case and control groups. The analysis of a typical GWAS dataset can be completed in less than a day on a standard workstation with parallel processing capability. The proposed framework was validated using simulated data and applied to real GWAS datasets using the Wellcome Trust Case Control Consortium (WTCCC) data. The results from simulated data showed the ability of iLOCi to identify various types of gene-gene interactions, especially for high-order interaction. From the WTCCC data, we found that among the top ranked interacting SNP pairs, several mapped to genes previously known to be associated with disease, and interestingly, other previously unreported genes with biologically related roles.

**Conclusion:**

iLOCi is a powerful tool for uncovering true disease interacting markers and thus can provide a more complete understanding of the genetic basis underlying complex disease. The program is available for download at http://www4a.biotec.or.th/GI/tools/iloci.

## Background

A major challenge for human genetics is identifying susceptibility genes for complex heritable diseases. Advanced single nucleotide polymorphism (SNP) genotyping technology and genome-wide association study (GWAS) are at the forefront of research in this area. In conventional single locus analysis, each variant is tested individually for disease association. Systematic analysis of GWAS data in this manner can typically uncover multiple SNPs associated with complex diseases [1-3]. These analyses have provided valuable insights into the genetics of complex diseases; however, they typically detect only common, low-risk variants each with small effect and explain only a tiny proportion of disease heritability [4].

The existence of interactions among genes (epistasis) has been proposed to constitute a major proportion of disease heritability, which is not captured by single-locus GWAS [5]. The genetical nature of epistasis can be described by several different models as shown in a variety of interaction schema discussed in [6]. Note that genetic factors primarily function through a complex mechanism; thus, epistatic interactions are not limited to independent gene pairs. Multiple genes interacting through a biological network (i.e. indirect interactions) exist which can modify disease penetrance and expressivity.

A number of methods for detecting epistatic interactions among genotypic data have been proposed. Most methods employ a statistical approach to identify interacting marker pairs based on deviation from a null distribution and estimation of type I error. These statistical approaches have been shown to work well in theory, e.g., regression methods [7,8], partitioning chi-square [9], Focused Interaction Testing Framework (FITF) [10], Bayesian model selection [11], and other recent approaches [12,13]. However, the need for control of type I error reduces power to detect interactions in real data, which is exacerbated by the huge number of statistical tests performed in this analysis [14].

Given the challenges for statistical approaches, non-statistical methods such as machine-learning and data-mining methods have been proposed for the study of genetic interactions [15,16]. Instead of model fitting, these methods attempt to explain all of the heritability in terms of marker interactions. Multifactor dimensionality reduction (MDR) is an brute-force method for identifying the most plausible interactions which fit the data [17]. However, MDR and other recently published exhaustive non-parametric approaches [18] are computationally complex and thus impractical for analysis of GWAS data. To overcome the computational burden of non-parametric analysis, several techniques have been developed that employ statistics to assist the non-parametric search for epistasis, including SNPHarvester [19], SNPRuler [20], and BOOST [21]. In these methods, the search space is reduced by a filtering step, usually employing a statistical threshold. The filtered dataset is then used for non-parametric search for epistasis. Although these methods can be applied for analysis of GWAS data, the interactions found rarely offer any new insights since the majority of interacting markers map to the same genomic regions. For example, the analysis of WTCCC (Wellcome Trust Case Control Consortium) data by BOOST revealed that after removal of linked pairs, no interactions were found for five of the seven diseases. Using another approach for exhaustive search of interactions, the most recent paper by Ueki and Tamiya [22] also reported very few interactions in the WTCCC data.

The possible reason for the disappointingly modest improvement of the current hybrid approaches is that they do not adequately account for marker dependencies not related to disease. A well known marker dependency which can confound the identification of genomic regions associated with disease is linkage disequilibrium (LD). LD is non-random association of genotypes at two or more loci that can be on the same or different chromosomes. LD is caused by a number of factors, including genetic linkage and the rate of recombination [23]. Earlier reports [24,25] showed that LD contrast, i.e., differences in LD patterns between case and control groups can reveal the disease signal above the noise of background LD in candidate disease regions. However, to our knowledge, LD contrast has not been employed for comprehensive genetic epistasis study, owing to the high computational complexity.

Clearly, a computationally efficient and comprehensive prioritization technique is required which accounts for marker dependencies unrelated to disease. Moreover, instead of trying to control type I error, a prioritization procedure may be more effective in revealing more of the true disease markers which may have modest individual effects and interact in complex higher-order networks.

In this paper, we propose a novel tool for prioritizing gene-gene interactions called iLOCi (interacting Loci). The iLOCi algorithm ranks all SNP pair combinations according to a novel heuristic that we call ρ_diff_. The iLOCi program is specifically designed to handle large-scale GWAS data partly through the application of data parallelization. The tests with WTCCC datasets show that the top ranked pairs by our algorithm reveal novel disease genes, several of which are consistent with biological networks underpining disease etiology.

## Methods

### iLOCi algorithm

**Figure 1:**
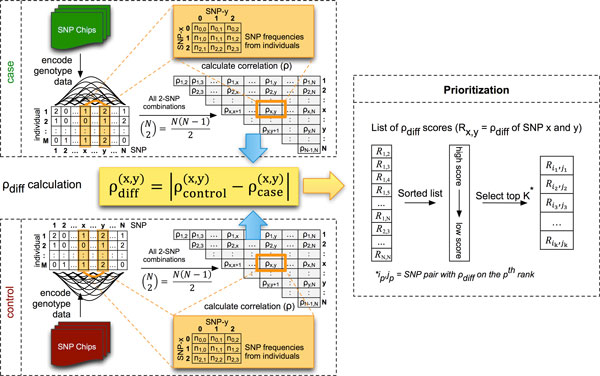
**The workflow of the iLOCi algorithm**.

The proposed iLOCi algorithm performs genome-wide analysis for identifying SNP pairs that are plausibly associated with a disease. No prior genetical assumptions are employed in the algorithm, which allows the exploration of different dimensions of the association results. The framework can be characterized into two main modules: 1) calculating SNP pair dependencies separately in case and control groups and 2) disease SNP pair prioritization as shown in Figure 1.

### Calculation of SNP pair dependencies

iLOCi explores all possible combinations of SNP pairs. Given *N *SNPs from a SNP array with the SNP index starting from 1 to *N*, there are a total of $\left(\begin{array}{c} N\\ 2 \end{array}\right)=\frac{N(N-1)}{2}$ possible pairs. Each SNP pair is assigned a unique index (*i*,*j*), where *i≠j*.

From the large number of SNP pairs, it is necessary to identify the dependency unrelated to disease. This dependency includes linkage disequilibrium (LD), population structure, genotype calling artifacts, etc. and is performed separately between the case and control groups. This step of the algorithm is called *dependence test*. Therefore, for each indexed SNP pair, the algorithm calculates two scores, *ρ_case _*and *ρ_control_*. The calculated *ρ *values using genotypic information were proven to be concordant with LD values (see Additional file 1). LD values are calculated using allelic deviation from the Hardy-Weinberg Equilibrium (HWE) model, which assumes that, without the introduction of specific disturbing factors, the frequencies of alleles and genotypes in a population remain constant from one generation to the next. However, it should be noted that the only information captured by *ρ *values is the correlation between markers, which is needed for identifying interactions. For LD calculation, the haplotypic phase is also considered, which is computationally very demanding for datasets of this size.

To compute marker *ρ *values, each SNP locus is considered as a discrete random variable and the numeric values of -1, 0 and 1 are assigned to homozygous wild (*w*), heterozygous (*h*), and homozygous variant (*v*) types respectively. This encoding ensures zero-means, which obviates a normalization step. Let *x *and *y *be two discrete random variables of SNP*x *and SNP*y*, respectively. Let *P*_(*x,y*) _represents a genotypic joint probability mass function, whose entries are the probability of genotype combinations from both SNPs. Hence, there are nine possible genotypic combinations that are represented by the following matrix:

$$P_{\left(x,y\right)}=\left[\begin{array}{ccc} P_{ww} & P_{wh} & P_{wv}\\ P_{hw} & P_{hh} & P_{hv}\\ P_{vw} & P_{vh} & P_{vv} \end{array}\right]$$

For example, *P_ww _*is a probability that (*x,y*) are both homozygous wild type. Each of these probabilities can be calculated by dividing the number of the joint genotypic outcomes with the total number of individuals for either case (*N*_case_) or control (*N*_control_) groups. For example, $P_{ww}^{\text{ctrl}}=P_{\left(x=w,y=w\right)}^{\text{ctrl}}=\frac{N_{\left(x=w,y=w\right)}^{ctrl}}{N_{\text{ctrl}}}$. The dependence test must be performed for all possible SNP pairs. The correlation value *ρ*_control _for each SNP pair is calculated as:

$$\begin{array}{l} =\frac{\left\{x_{w}y_{w}P_{ww}^{\text{ctrl}}+x_{w}y_{h}P_{wh}^{\text{ctrl}}+x_{w}y_{v}P_{wv}^{\text{ctrl}}\right\}+\left\{x_{h}y_{w}P_{hw}^{\text{ctrl}}+x_{h}y_{h}P_{hh}^{\text{ctrl}}+x_{h}y_{v}P_{hv}^{\text{ctrl}}\right\}+\left\{x_{v}y_{w}P_{vw}^{\text{ctrl}}+x_{v}y_{h}P_{vh}^{\text{ctrl}}+x_{v}y_{v}P_{vv}^{\text{ctrl}}\right\}}{\left(x_{w}^{2}P_{x=w}^{\text{ctrl}}+x_{h}^{2}P_{x=h}^{\text{ctrl}}+x_{v}^{2}P_{x=v}^{\text{ctrl}})\times (y_{w}^{2}P_{y=w}^{\text{ctrl}}+y_{h}^{2}P_{y=h}^{\text{ctrl}}+y_{v}^{2}P_{y=v}^{\text{ctrl}}\right)}\\ =\frac{\left\{(-1)(-1)P_{ww}^{\text{ctrl}}+(-1)(0)P_{wh}^{\text{ctrl}}+(-1)(1)P_{wv}^{\text{ctrl}}\right\}+\left\{(0)(-1)P_{hw}^{\text{ctrl}}+(0)(0)P_{hh}^{\text{ctrl}}+(0)(1)P_{hv}^{\text{ctrl}}\right\}+\left\{(1)(-1)P_{vw}^{\text{ctrl}}+(1)(0)P_{vh}^{\text{ctrl}}+(1)(1)P_{vv}^{\text{ctrl}}\right\}}{\left({\left(-1\right)}^{2}P_{x=w}^{\text{ctrl}}+{\left(0\right)}^{2}P_{x=h}^{\text{ctrl}}+{\left(1\right)}^{2}P_{x=v}^{\text{ctrl}})\times ({\left(-1\right)}^{2}P_{y=w}^{\text{ctrl}}+{\left(0\right)}^{2}P_{y=h}^{\text{ctrl}}+{\left(1\right)}^{2}P_{y=v}^{\text{ctrl}}\right)}\\ =\frac{P_{ww}^{\text{ctrl}}-P_{wv}^{\text{ctrl}}-P_{vw}^{\text{ctrl}}+P_{vv}^{\text{ctrl}}}{\left(P_{x=w}^{\text{ctrl}}+P_{x=v}^{\text{ctrl}})\times (P_{y=w}^{\text{ctrl}}+P_{y=v}^{\text{ctrl}}\right)} \end{array}$$

Note that $P_{x=w}^{\text{ctrl}},P_{x=v}^{\text{ctrl}},P_{y=w}^{\text{ctrl}},P_{y=v}^{\text{ctrl}}$ are the estimated probability of SNPx wild type, SNPx variant type, SNPy wild type and SNPy variant type respectively.

By the same reasoning, *ρ*_case _is calculated as:

$$=\frac{P_{ww}^{\text{case}}-P_{wv}^{\text{case}}-P_{vw}^{\text{case}}+P_{vv}^{\text{case}}}{\left(P_{x=w}^{\text{case}}+P_{x=v}^{\text{case}})\times (P_{y=w}^{\text{case}}+P_{y=v}^{\text{case}}\right)}$$

#### Disease SNP pair prioritization

The next step is to identify whether the same SNP pair (*x*,*y*) from case and control groups have contrasting patterns of *ρ *values. A *difference test *is performed by differentiating the *ρ *values between the case and control groups using a simple subtraction operation, namely *ρ*_diff_=|*ρ*_control_-*ρ*_case_|.

To select the highly associated SNP pairs, all SNP pairs are ranked according to the *ρ*_diff _values. The ranking of top SNP pairs was chosen, rather than a *P-*value cutoff in order to avoid too many false positive pairs due to the heavy-tailed distribution phenomenon, where the Gaussian distribution decreases faster than the distribution of disease associated SNP pairs [26].

### Parallel computing algorithm implemented in iLOCi

The iLOCi algorithm is designed for genome-scale analysis which requires the computation of a huge number of SNP interaction pairs, e.g.≈1.25x10^11 ^pairs for a 500,000 SNP dataset. Data parallelization is applied to accelerate this computationally intensive and time-consuming process. The SNP interaction matrix is divided into submatrices of 100,000 or fewer SNPs each. Each SNP interaction submatrix is computed in parallel using a MacPro workstation with 2×2.4 GHz quad-core Intel Xeon processors with 8GB RAM. With this configuration, the complete WTCCC dataset can be analyzed in 19 hours. Details for implemention of the code and data parallelization are available upon request.

### Testing iLOCi algorithm performance using simulated data

The performance of iLOCi for detecting disease-associated gene interactions was evaluated and compared with FastEpistasis [27]. The evaluation was made using simulated datasets, which were generated using the GenomeSIM program [28]. The algorithm performance was determined for detection of four different epistatic interaction scenarios:

1) Single pair interaction without marginal effects: Eighteen epistatic models in [29] with heritability (h^2^) of 0.2, 0.3, and 0.4 were used for performance comparison (see Additional file 2: Table S1). These heritability levels were chosen to represent those typically found in common complex diseases. The minor allele frequency (MAF), which is the frequency of the less common allele, was assigned to be two levels, 0.2 and 0.4. In total, there are six model groups comprising three models with the same heritability and MAF for each group. 100 independent datasets containing 1600 samples (800 cases and 800 controls) with 100 SNPs were generated for each model group.

2) Single pair interaction with marginal effects: Six epistatic models in [30] with MAF of 0.5 were tested (see Additional file 2: Table S2). 100 independent datasets containing 800 samples (400 cases and 400 controls) and 100 independent datasets containing 1600 samples (800 cases and 800 controls) with 100 SNPs each were generated for each model group.

3) Multiple independent interacting pairs without marginal effects: Eight models of multiple interactions described in supplementary material of [19] were tested. Each of these models were generated from five epistatic models described in [29]. Each model used the same heritability and MAF. 100 independent datasets containing 1600 samples (800 cases and 800 controls) and 100 SNPs were generated for each model group.

4) Higher-order interactions: Data were simulated for the eight interaction network models based on pairwise interaction described in [31] for three-, four-, and five-loci interating networks (see Additional file 2: Table S3). 100 independent datasets containing 800 samples (400 cases and 400 controls) were generated. The number of SNPs varies from model to model.

The algorithm performance was demonstrated by the percentage of accuracy, which is determined by the proportion of 100 independent datasets in which the algorithm correctly identified the interacting SNP pairs. For situations 1 and 2, the identification of disease SNP pair is defined as correct if the disease SNP pair is the top ranked pair with the highest ρ_diff _score (for iLOCi) or the lowest *P*-value (for FastEpistasis). For multiple independent interacting pairs (case 3), the identification is taken as correct when all five disease SNPs fall in the top five ranked pairs with highest ρ_diff _score (for iLOCi) or lowest *P*-value (for FastEpistasis). The prediction of higher-order interactions is defined as correct when all disease SNPs are found within all top ranked pairs. The top ranked pairs are defined as all consecutive pairs comprising at least one disease SNP in each pair.

### Testing algorithm performance using the WTCCC dataset

In addition to the simulated data, our algorithm was applied to the real genotypic data of WTCCC (Wellcome Trust Case Control Consortium) [3]. This dataset encompasses ~500,000 SNP genotypic data of ~17,000 British samples which are divided into 3000 shared control samples and ~2000 case samples for each of seven complex diseases: bipolar disorder (BD), coronary artery disease (CAD), Crohn's disease (CD), hypertension (HT), rheumatoid arthritis (RA), type1 (T1D) and type2 (T2D) diabetes.

For these real datasets, data cleaning was required prior to the analysis. We considered only SNPs and individuals passing WTCCC data quality control [3]. We further filtered the SNP set using MAF>0.05 leaving 355,882 SNPs (complete set) for all diseases. We also generated a SNP marker gene-only subset of 176,148 present in genes (defined as within 10Kb flanking an annotated gene model reported in RefSeq version 36.3).

First, ρ_diff _values for the seven WTCCC diseases were calculated for all possible (≈63×10^9 ^for complete and ≈15×10^9 ^for the gene-only subset) pairs. Next, the empirical ρ_diff _distributions for each disease were graphed using kernel density plot. For the gene-only SNP subset analysis, the top ranked 1000 SNP pairs were chosen for functional analysis to uncover biological significance. From these pairs, a list of genes was extracted based upon RefSeq (version 36.3) physical locations of SNPs in the genome. To understand the biological significance of the novel genes reported by our algorithm, we also used the candidate gene prioritization feature of ToppGene [32] using the cutoff of *P*-value = 0.01 with Bonferroni correction. The training sets for the ToppGene candidate gene prioritization were the lists of all genes reported in the HuGE Navigator database [33] for the seven diseases. The test sets for the ToppGene analysis were the lists of novel (not reported in HuGE Navigator database) genes represented among the top ranked 1000 SNP pairs obtained from iLOCi.

## Results

### iLOCi algorithm validation

We used simulated datasets to validate the iLOCi algorithm for identifying various disease-associated epistatic interactions. We chose FastEpistasis for performance comparison with iLOCi due to the fact that the data were simulated according to an interaction model; hence this tool would be most suitable for testing. Moreover, the theoretical basis for FastEpistasis is widely accepted for genome-wide analysis.

**Figure 2:**
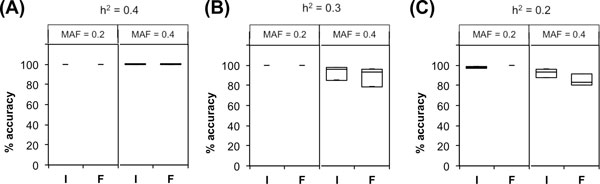
**The performance comparison between iLOCi (I) and FastEpistasis (F) on epistatic models without marginal effects**. The algorithm performance is shown as the percentage of accuracy, which is the number of simulated datasets (out of 100) in which the correct SNP pair is identified. The accuracy was tested for two different MAF (0.2, 0.4) and three different levels of heritability (A) 0.4, (B) 0.3, and (C) 0.2.

**Figure 3:**
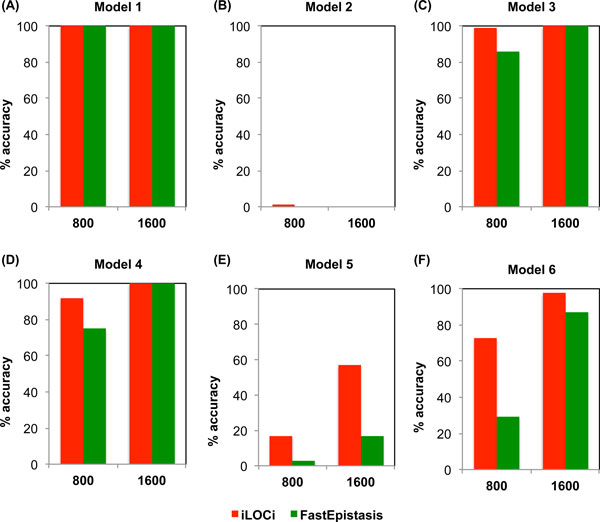
**The performance comparison between iLOCi and FastEpistasis on epistatic models with marginal effects**. The percentage of accuracy is shown for two different sample sizes (800 and 1600) for six different pairwise interaction models (A-F).

**Figure 4:**
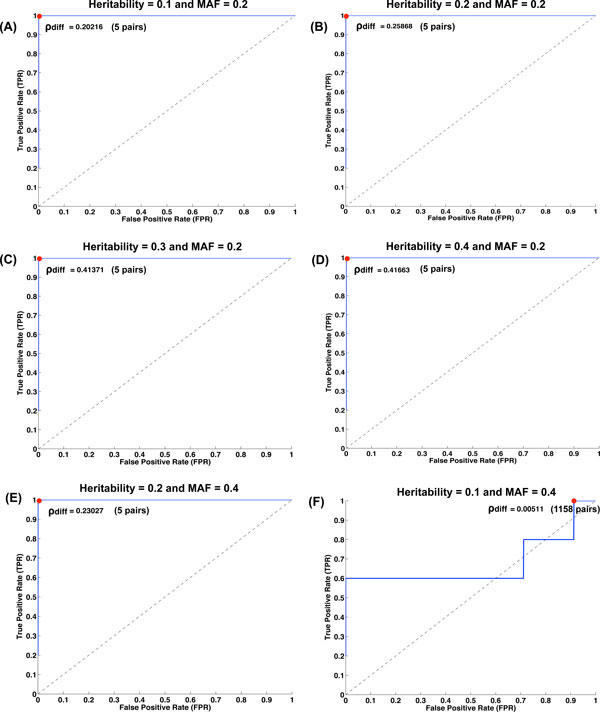
**The Receiver Operating Characteristic (ROC) curves for simulation datasets of hybrid models**. The ROC curves are displayed for five independent interacting SNP pairs.The MAF and heritability parameters were varied (A) h^2^=0.1, MAF = 0.2, (B) h^2^=0.2, MAF = 0.2, (C) h^2^=0.3, MAF = 0.2, (D) h^2^=0.4, MAF = 0.2, (E) h^2^=0.2, MAF = 0.4, (F) h^2^=0.1, MAF = 0.4. The ρ_diff_values are shown that give the maximum true positive rate with the lowest false positive rate (red dots).

The first result testing for a single interacting pair demonstrated that the top ranked iLOCi pair was the disease interacting pair in 18 different inheritance models without the presence of marginal effects. Overall, its performance was approximately the same as FastEpistasis for most of the model groups and slightly better in some cases (h^2^=0.2, MAF = 0.4; h^2^=0.3, MAF = 0.4) as shown in Figure 2. For epistatic interactions with marginal effects, iLOCi outperformed FastEpistasis in most models, except in model 2 for which both methods failed to detect the interacting disease marker pair (Figure 3). Furthermore, we want to demonstrate the specificity as well as sensitivity of iLOCi for detecting multiple interacting disease marker pairs as would be present in a real dataset. Therefore, the receiver operating characteristics (ROC) were plotted for different thresholds of ranked marker pairs, and for different models of heritability and MAF (Figure 4). Generally, iLOCi has high sensitivity and specificity, although the performance tends to be worse with lower degrees of heritability. Moreover, it should be noted that the minimum ρ_diff _scores that give 100% sensitivity vary greatly from 0.00511 to 0.41663.

**Figure 5:**
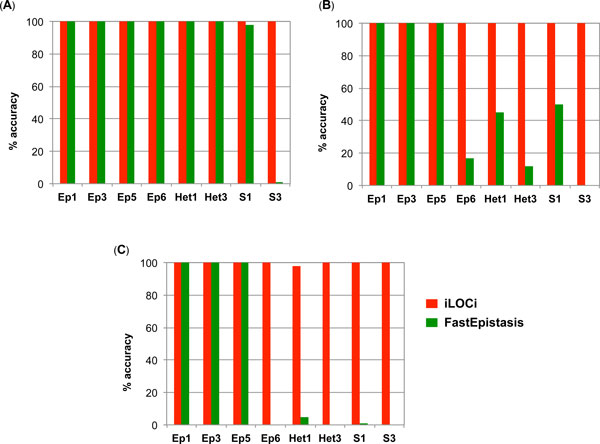
**The performance comparisons between iLOCi and FastEpistasis on high-order interaction models**. The percentage of accuracy is shown for different models (Ep1, Ep3, Ep5, Ep6, Het1, Het3, S1, S3) of high-order interactions among (A) three-loci, (B) four-loci, and (C) five-loci.

In addition to independent interacting pairs, we examined the ability of iLOCi and FastEpistasis to detect higher-order interactions of 3, 4, and 5 loci disease interaction networks for eight models at each level (Figure 5). iLOCi can detect all eight models for all levels of interactions; however, FastEpistasis failed to identify all S3 model interactions. Furthermore, FastEpistasis could detect, with higher than 50% accuracy, in fewer than 50% of the 4-loci network models and only Ep1, Ep3 and Ep5 of the 5-loci network models.

In conclusion, these experiments with simulated data validated the iLOCi algorithm for identifying all four types of higher-order gene interaction. iLOCi performance was comparable to FastEpistasis for a variety of two-locus interaction models; however, iLOCi was markedly superior for detecting high-order interactions. This would be a major advantage of iLOCi for analysis of real data since high-order interaction is the type of interaction likely to be found in real data of complex diseases and may account for current missing heritability.

### iLOCi analyses of WTCCC data

**Figure 6:**
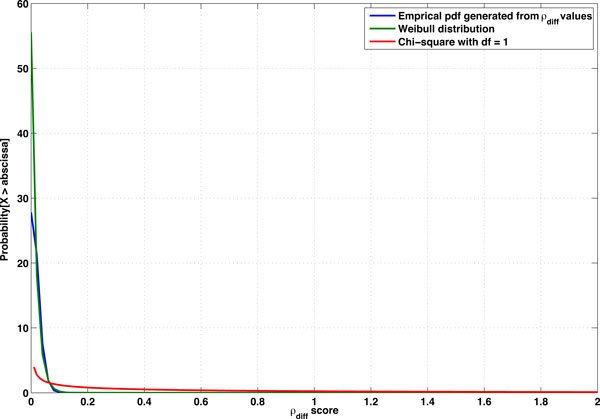
**The frequency distribution of ρ_diff _values from WTCCC datasets**. The plot shows the empirical probabilty density function (pdf) generated from combined ρ_diff_values from all seven diseases of WTCCC datasets.The pdf plots generated from each disease are indistinguishable from combined pdf. The plots for Weibull distribution (k = 1, λ=0.018) and Chi-square distribution (degree of freedom = 1) are shown in the same axes.

The iLOCi algorithm was tested against real data obtained from WTCCC. The distribution of ρ_diff _values follows a Weibull distribution pattern for all seven diseases (Figure 6). From the Weibull distribution with k = 1 and λ=0.018, we calculated *P*-values for ρ_diff _scores ranging from 0.05 to 1.0 (see Table 1). For the seven diseases, we selected the top 1000 pairs for which the calculated minimum *P*-values vary from <2.22e-16 to 1.14e-7 in complete SNP set analysis, and from <2.22e-16 to 4.72e-5 in gene-only SNP analysis (see Table 2).

**Table 1:** The lookup table of *P*-values for the associated ρ_diff _scores

ρ_diff_score	*P*-value
0.05	6.2177e-2
0.10	3.8659e-3
0.15	2.4037e-4
0.20	1.4945e-5
0.25	9.2925e-7
0.30	5.7777e-8
0.35	3.5924e-9
0.40	2.2336e-10
0.45	1.3888e-11
0.50	8.6353e-13
0.55	5.3735e-14
0.60	3.3307e-15
0.65	2.2204e-16
0.70	<2.2204e-16
0.75	<2.2204e-16
0.80	<2.2204e-16
0.85	<2.2204e-16
0.90	<2.2204e-16
0.95	<2.2204e-16
1.00	<2.2204e-16

The *P*-values were calculated based on the fitted Weibull distribution with k = 1 and λ=0.018.

**Table 2:** The ρ_diff _scores of the 1^st ^and 1000^th ^ranked SNP pairs and their associated *P*-values

Complete set of SNPs (355882 SNPs)					
**Disease**	**1^st^ρ_diff_**	**1^st^*P*-value**	**1000^th^ρ_diff_**	**1000^th^*P*-value**	**Avg. ρ_diff_± SD**

BD	0.2878	1.1410e-7	0.2680	3.4206e-7	0.2718**±**0.0035
CAD	0.9317	<2.2204e-16	0.9132	<2.2204e-16	0.9171**±**0.0031
CD	0.3085	3.6109e-8	0.2849	1.3351e-7	0.2887**±**0.0034
HT	0.2834	1.4510e-7	0.2626	4.6022e-7	0.2667**±**0.0037
RA	0.9042	<2.2204e-16	0.8866	<2.2204e-16	0.8903**±**0.0031
T1D	1.0731	<2.2204e-16	0.9996	<2.2204e-16	1.0040**±**0.0056
T2D	0.3338	8.8226e-9	0.2159	6.1867e-6	0.2198**±**0.0052

**Gene-only SNPs (176148 SNPs)**					

**Disease**	**1^st^ρ_diff_**	**1^st^*P*-value**	**1000^th^ρ_diff_**	**1000^th^*P*-value**	**Avg. ρ_diff_± SD**

BD	0.2447	1.2445e-6	0.2224	4.2957e-6	0.2259**±**0.0032
CAD	0.9294	<2.2204e-16	0.9102	<2.2204e-16	0.9143**±**0.0035
CD	0.2653	3.9790e-7	0.2248	3.7769e-6	0.2280**±**0.0033
HT	0.1793	4.7229e-5	0.1561	1.7142e-4	0.1605±0.0043
RA	0.9040	<2.2204e-16	0.8832	<2.2204e-16	0.8875**±**0.0036
T1D	1.0731	<2.2204e-16	0.9957	<2.2204e-16	1.0007**±**0.0061
T2D	0.3338	8.8226e-9	0.2127	7.3731e-6	0.2168**±**0.0052

The highest and the lowest ρ_diff _scores including their associated *P*-values are displayed with the average scores of top 1000 SNP pairs from the analyses of WTCCC.

From iLOCi analysis using the complete SNP marker set, it was found that the great majority of the SNPs have not been previously reported to be associated with the diseases [3]. Furthermore, the majority of these SNPs also do not map to annotated genes. The list of top 1000 SNP pairs is available in Additional File 3. For each disease, iLOCi identified 'hub' SNPs, i.e. SNPs that pair with many other SNPs, e.g., rs1553460 pairs with 1000 other SNPs in BD (Table 3).

**Table 3:** The hub SNPs/genes identified in the top-ranked 1000 SNP pairs

Hub SNPs from analyses of complete SNP set		
**Disease**	**Hub SNPs (Genomic position)**	**# Interacting SNPs**

BD	rs1553460 (Chr4:17804959)	1000

CAD	rs3785579 (Chr17:62472963)	1000

CD	rs1553460 (Chr4:17804959)	978

	rs4471699 (Chr16:30227808)	22

HT	rs10843660 (Chr12:30259724)	999

RA	rs3785579 (Chr17:62472963)	1000

T1D	rs9273363 (Chr6:32734250)	1000

T2D	rs7077039 (Chr10:114779067)	833

	rs10787472 (Chr10:114771287)	54

	rs11196208 (Chr10:114801306)	39

	rs11196205 (Chr10:114797037)	30

	rs10885409 (Chr10:114798062)	22

	rs4074720 (Chr10:114738487)	17

**Hub genes from gene-only SNP analyses**		

**Disease**	**Hub genes**	**# Interacting genes**

BD	*CENPN*: centromere protein N	653

CAD	*CACNG1*: calcium channel, voltage-dependent, gamma subunit 1	709

CD	*ATG16L1*: ATG16 autophagy related 16-like 1 (*S. cerevisiae*)***	256

	*IL23R*: interleukin 23 receptor ***	20

HT	*tcag7.23*: similar to ribosomal protein L18; 60S ribosomal protein L18	170

	*BCAT1*: branched chain aminotransferase 1, cytosolic ***	57

	*SAMD4A*: sterile alpha motif domain containing 4A *	27

	*GAB1*: GRB2-associated binding protein 1 *	25

	*RHOJ*: ras homolog gene family, member J	20

	*LYPD5*: LY6/PLAUR domain containing 5 *	12

RA	*CACNG1*: calcium channel, voltage-dependent, gamma subunit 1	676

T1D	*HLA-DQB1*: major histocompatibility complex, class II, DQ beta 1**	686

T2D	*TCF7L2*: transcription factor 7-like 2 (T-cell specific, HMG-box)***	481

* Genes associated with disease SNPs that were previously reported in WTCCC original paper

** Genes previously reported to be disease-associated in HuGE Navigator database

*** Genes previously reported to be disease-associated in both WTCCC paper and HuGE Navigator database

**Figure 7:**
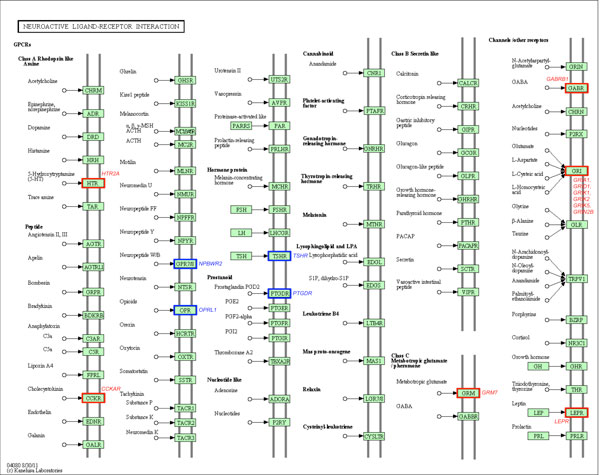
**The iLOCi detected genes from BD and their maps in 'neuroactive ligand-receptor interaction' pathway**. The KEGG pathway diagram [34] shows the mapping of BD-associated genes identified among 1000 top ranked iLOCi pairs in 'neuroactive ligand-receptor interaction' KEGG pathway. The gene families containing the genes previously reported in HuGE Navigator database and the novel disease genes are highlighted in the red boxes and the blue boxes, respectively, with their associated gene names.

**Figure 8:**
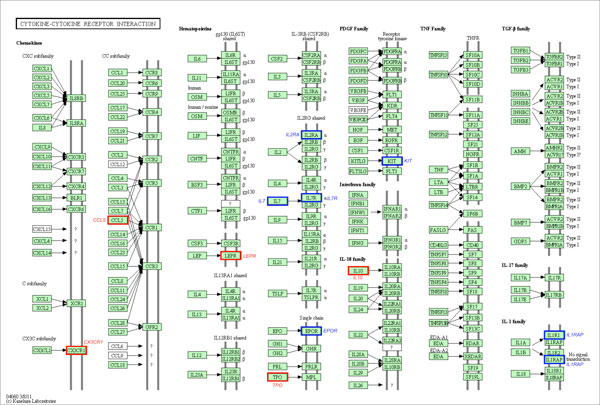
**The iLOCi detected genes from CAD and their maps in 'cytokine-cytokine receptor interaction' pathway**. The KEGG pathway diagram [34] shows the mapping of CAD-associated genes identified among 1000 top ranked iLOCi pairs in 'cytokine-cytokine receptor interaction' pathway. The gene families containing the genes previously reported in the HuGE Navigator database and novel disease genes are highlighted in the red boxes and the blue boxes, respectively, with their associated gene names.

**Figure 9:**
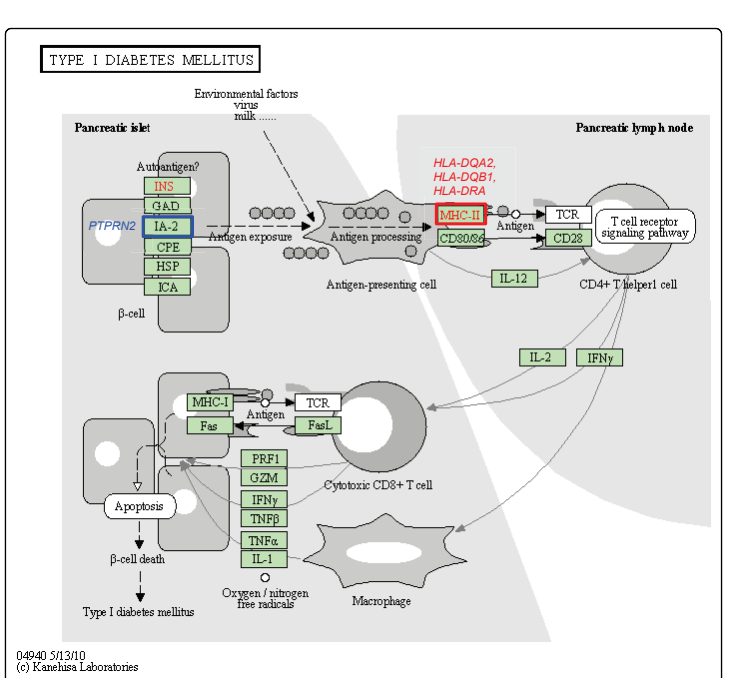
**The iLOCi detected genes from T1D and their maps in 'type I diabetes mellitus' pathway**. The KEGG pathway diagram [34] shows the mapping of T1D-associated genes identified among 1000 top ranked iLOCi pairs in the 'type I diabetes mellitus' KEGG pathway. The gene families containing the genes previously reported in HuGE Navigator database and the novel disease genes are highlighted in the red boxes and the blue boxes, respectively, with their associated gene names.

Owing to the fact that the majority of interacting SNPs do not map to annotated genes, we re-analyzed the data using the gene-only SNP subset. 'Hub' SNPs were also observed at the gene level (Table 3). From this analysis, it was noted that the top ranked 1000 SNP pairs of all seven diseases map to 321 disease-gene associations that have been annotated on the HuGE Navigator database (see Table 4, Additional File 4). On the other hand, the majority of the disease interacting genes among these pairs reported by iLOCi are novel. Moreover, most of these genes were not reported in the original WTCCC study (Table 4). To evaluate the biological significance of the novel genes among these pairs, the ToppGene candidate gene prioritization tool was employed. The full results are shown in Additional Files 3 and 4. Among the novel genes identified by iLOCi, it was observed that some well known disease pathways from KEGG [34] contain several of these genes (see Additional File 5). For instance, the 'neuroactive ligand-receptor interaction' pathway in BD contains 4 novel genes in addition to 11 previously reported genes (Figure 7). Other prominent disease pathways include 'cytokine-cytokine receptor interaction' for CAD (Figure 8) and 'type I diabetes mellitus' for T1D (Figure 9).

**Table 4:** The disease association of iLOCi selected genes from gene-only SNP analyses

Disease	# iLOCi genes in top 1000 SNP pairs	Reported in WTCCC single SNP analyses		Reported in HuGE Navigator database	
		
		# Analyzed genes (# SNPs)	# iLOCi genes	# Analyzed genes (# SNPs)	# iLOCi genes
BD	654	42 (1757)	8	665 (16598)	52
CAD	710	29 (2097)	3	735 (11564)	37
CD	279	54 (1651)	4	531 (7181)	10
HT	595	32 (3164)	19	1240 (22004)	64
RA	677	34 (822)	4	503 (5902)	19
T1D	687	39 (1153)	5	512 (6924)	29
T2D	486	29 (1289)	5	2456 (41244)	110

The table displays the number of previously reported disease-associated genes which were found in all analyzed genes and in the set of genes involved in top 1000 interaction pairs. The reported disease genes are shown for both the genes associated with disease SNPs from WTCCC paper [3] and the ones reported in HuGE Navigator database [33].

## Discussion

In this study, we have developed a new pairwise SNP-interaction prioritization algorithm for GWAS. We hypothesized that by first accounting for pairwise marker dependencies among case and control groups, it would be possible to observe true disease interactions above the noise of dependent markers unrelated to disease, as was proposed in earlier studies of LD contrast (see Background).

In GWAS data, it is well known that LD generates strong pairwise dependency signals that are used to identify disease associated SNPs by imputation. However, this type of signal predominates pairwise markers in analysis of gene interactions. For example, in the approach used by Wan et al. [21], the majority of the interactions identified for all seven WTCCC datasets can be attributed to LD effect, i.e., the interacting SNPs are within 1Mb of each other in the same genomic region. To validate our approach correcting for pairwise dependencies unrelated to disease SNP interactions, extensive tests were performed on simulated data. For a simple model with only one interacting pair, the top ranked iLOCi pair is correctly identified as the disease marker pair. When testing for multiple interacting pairs, iLOCi has high accuracy under the conditions of high heritability and informativeness, i.e., low MAF. On the other hand, low heritability and/or informativeness leads to type I error as observed by ROC plot. In general, the ρ_diff _scores reflect the degree of heritability and informativeness. Hence, it is not possible to use a single ρ_diff _cutoff for identifying disease interactions in the real case when the heritability and informativeness are unknown.

From analyses of real GWAS data, it was found that the ρ_diff _distributions for all seven diseases could be represented by a single kernel density function with Weibull distribution. However, the range of ρ_diff _values varies among the diseases and follow the known heritability pattern, i.e., HT has the lowest heritability and lowest top ρ_diff _score, while T1D has the highest heritability and highest top ρ_diff _score (Table 2). Although it is possible to calculate *P*-values of the interacting pairs and use them as cutoffs for prioritization, we consider the use of *P*-value cutoffs inappropriate. For example, a *P*-value of 1e-5 (corresponding to ρ_diff _values of approximately 0.2 or greater) would give approximately 16 million significant pairs for T1D and 200,000 pairs for HT. The same phenomenon of unacceptable type I error was found by others when using FastEpistasis for analysis of real datasets. It is debatable whether Bonferroni correction is valid since the tests are not independent, as shown by the heavy-tailed distributions of ρ_diff _. Current methods for correction of type I error by false discovery rate are also likely to be impractical because of the requirement for permutation testing.

Instead of using *P*-value significance thresholds, we used the top ranked 1000 SNP pairs for prioritization, which account for a very small portion (<0.0001%) of all possible pairs. Rather than attempting to identify all gene interactions, which practically can not be found [35], we limit the prioritization to the top ranked pairs that are most likely to contain the genetic interactions which are informative of the disease etiology, i.e., disease pathways. From the full SNP set analysis, several hub SNPs were identified for each disease which interact with many other SNPs. For some diseases such as T1D, these hub SNPs map to well-known disease associated genes. However, hub SNPs for BD, HT, and CD do not map to genes. These hub SNPs may mediate interactions at an unknown gene regulatory level, e.g. as non-coding RNAs, miRNAs or cis-regulatory elements. Since our knowledge of gene regulation is far from complete [36], we repeated the iLOCi analysis on the gene-only SNPs subset. By restricting the analysis to SNP pairs in genes only, the ToppGene systems approach for gene prioritization was appropriate, as used by others for GWAS data [37-39].

Gene-based prioritization of the interacting SNP pairs revealed significant representation of previously described disease associated genes. Therefore, we are confident that the novel genes found among the prioritized SNP pairs are novel disease-associated genes. For each disease, hub genes were found which pair with many other genes. Some of these disease hub genes are known and have been replicated as disease genes by conventional single-SNP GWAS, including the MHC gene *HLADQB1 *for T1D and *TCF7L2 *for T2D. However, some hub genes have not been reported previously, e.g. the *CACNG1 *gene for RA. This gene's SNP shows a modest *P*-value (>1e-4) for association by single SNP analysis [3]; therefore, the disease association of this SNP is dependent on multiple interactions with other loci. For each disease, including those with low heritability such as HT, we are able to suggest novel genes and pathways for further investigation, including re-analysis of other GWAS datasets for the same diseases.

## Conclusions

In this article, we introduce a novel SNP interaction prioritization method, called iLOCi. The algorithm is computationally efficient, and thus suitable for exhaustive search for interactions along markers in a typical GWAS dataset. We have shown that the approach taken by iLOCi in which marker dependencies unrelated to disease are accounted for reveal genetic interactions of biological relevance to complex disease.

## Competing interests

The authors declare that they have no competing interests.

## Authors' contributions

JP designed the algorithm and the experiments, generated simulated data, analyzed test results, and wrote the manuscript. CN performed most experiments on simulated and real datasets. AI designed the algorithm and performed the mathemathical proof of formula. SK implemented iLOCi program. AA designed the algorithm. CB performed the functional analysis of real dataset. PJS wrote the manuscript and discussed the test results. ST designed the algorithm, discussed the test results, and wrote the manuscript.

## Supplementary Material

Additional file 1 **The mathematical details of ρ_diff _value and its relation with LD (iLOCi_details.pdf)**. This file includes the mathematical details of iLOCi formula and its relationship with the allele-based LD calculation. Click here for file

Additional file 2 **Penetrance tables for dataset simulation (Penetrance_tables.pdf)**. This file includes the penetrance models used for dataset simulation of two-locus and high-order ineractions. Click here for file

Additional file 3 **Top 1000 SNP pairs from analyses of complete SNP set of WTCCC (TopPairs_Complete.xls)**. This file includes the list of top 1000 SNP pairs with their associated genes obtained from the iLOCi analyses of all SNPs passing the quality control step. The evidences for disease association of each identified gene as reported in WTCCC original paper and HuGE Navigator database are also shown. The genes identified as candidate disease genes from ToppGene prioritization are indicated with their rank numbers and *P*-values. Click here for file

Additional file 4 **Top 1000 SNP pairs from analyses of gene-only SNP set of WTCCC (TopPairs_GeneOnly.xls)**. This file includes the list of top 1000 SNP pairs with their associated genes obtained from the iLOCi analyses of gene-only SNPs. The evidences for disease association of each identified gene as reported in WTCCC original paper and HuGE Navigator database are also shown. The genes identified as candidate disease genes from ToppGene prioritization are indicated with their rank numbers and *P*-values. Click here for file

Additional file 5 **Pathway enrichment analysis of WTCCC datasets (Pathway_analysis.xls)**. This file includes the list of enriched biological pathways obtained from ToppGene program using the training sets of HuGE Navigator disease-associated genes. The pathway *P*-value is reported along with the list of iLOCi identified genes associated with such pathway. For each pathway, the number of genes previously reported in HuGE Navigator database, reported in WTCCC paper, and the novel disease genes, is shown. Click here for file
