# Supplementary material for: iLOCi: a SNP interaction prioritization technique for detecting epistasis in genome-wide association studies
Source: BMC Genomics. 2012 Dec 7;13(Suppl 7):S2. doi: 10.1186/1471-2164-13-S7-S2 (PMC3521387; doi:10.1186/1471-2164-13-S7-S2)
Supplement: Additional file 1 — The mathematical details of ρdiff value and its relation with LD (iLOCi_details.pdf). This file includes the mathematical details of iLOCi formula and its relationship with the allele-based LD calculation. [file 1471-2164-13-S7-S2-S1.pdf]

The mathematical relationship between ρ_{diff} value calculated using genotype information and LD calculated from allelic information

Let a and b be random variables for alelle from loci A and B where $a \in \{-1, 1\}$$ and $b \in \{-1, 1\}$ and the observed haplotype frequency is shown in Table 1.

Table 1: Two haplotype observed frequency

	A_1	A_2
B_2	x_{21}	x_{22}

We denote alelle frequency from loci A and B as in Table 2.

Table 2: Estimated allele frequency

A_1	A_2	B_1	B_2
p_1	p_2	q_1	q_2

Given that the loci A and B are independent, expected haplotype frequency is determined by

$$x_{11} = p_1 q_1$$

$$x_{12} = p_1 q_2$$

$$x_{21} = p_2 q_1$$

$$x_{22} = p_2 q_2$$

Linkage disequilibium (LD) is defined as the deviation of the expected haplotype frequency and the definition of LD is given in Table 3.

Table 3: Two observed haplotye frequency

	A_1	A_2
B_1	$x_{11} = p_1 q_1 + D$	$x_{21} = p_2 q_1 - D$
B_2	$x_{12} = p_1 q_2 - D$	$x_{22} = p_2 q_2 + D$

Let us compute the covariance of haplotype from two locus. The covariance is defined by

$$Cov(a, b) = E[ab] - E[a]E[b]$$

From Table 1, $E[ab]$ calculation is shown in the following equation:

$$\begin{aligned} E[ab] &= -1 \times -1 \times x_{11} + -1 \times 1 \times x_{21} + 1 \times -1 \times x_{12} + 1 \times 1 \times x_{22} \\ &= x_{11} - x_{21} - x_{12} + x_{22} \end{aligned}$$

and $E[a]E[b]$ is given by

$$\begin{aligned} E[a]E[b] &= (-1 \times p_1 + 1 \times p_2)(-1 \times q_1 + 1 \times q_2) \\ &= p_1 q_1 - p_1 q_2 - p_2 q_1 + p_2 q_2 \end{aligned}$$

It follows that the haplotype covariance is

$$\begin{aligned} E[ab] - E[a]E[b] &= x_{11} - x_{21} - x_{12} + x_{22} - (p_1 q_1 - p_1 q_2 - p_2 q_1 + p_2 q_2) \\ &= (x_{11} - p_1 q_1) - (x_{21} - p_2 q_1) - (x_{12} - p_1 q_2) + (x_{22} - p_2 q_2) \end{aligned}$$

From the relationship between observed haplotype frequency and LD in Table 3, we have

$$\begin{aligned} E[ab] - E[a]E[b] &= (x_{11} - p_1 q_1) - (x_{21} - p_2 q_1) - (x_{12} - p_1 q_2) + (x_{22} - p_2 q_2) \\ &= D - (-D) - (-D) + D \\ &= 4D \end{aligned}$$

which is the relationship between covaraince and LD.

Next, we consider genotype correlation. Let S_1 and S_2 be random variables of SNP1 and SNP2 respectively with the values of $S_1 \in \{-1, 0, 1\}$ and $S_2 \in \{-1, 0, 1\}$. The observed genotype frequency is given Table 4.

Table 4: Genotype observed frequency

	A_1A_1	A_1A_2	A_2A_2
B_1B_2	f_{21}	f_{22}	f_{23}
B_2B_2	f_{31}	f_{32}	f_{33}

If two genotype locus are independent, the expected frequencies are given in Table 5.

Table 5: Genotype expected frequency

	A_1A_1	A_1A_2	A_2A_2
B_1B_1	$p_1^2q_1^2$	$2p_1q_1^2$	$p_2^2q_2^2$
B_1B_2	$2p_1^2p_1q_1$	$4p_1p_2q_1q_2$	$2p_2^2q_1q_2$
B_2B_2	$p_1^2q_2^2$	$2p_1p_2q_2^2$	$p_2^2q_2^2$

Since $S_1, S_2 \in \{-1, 0, 1\}$, the covariance of two SNPs is computed by

$$\begin{aligned} E[S_1S_2] &= -1\times-1\times f_{11}+-1\times 1\times f_{13}+1\times-1\times f_{31}+1\times 1\times f_{33} \\ &= f_{11}-f_{13}-f_{31}+f_{33} \end{aligned}$$

and

$$\begin{aligned} E[S_1]E[S_2] &= (-1\times p_1^2+1\times p_2^2)(-1\times q_1^2+1\times q_2^2 \\ &= p_1^2q_1^2-p_2^2q_1^2-p_1^2q_2^2+p_2^2q_2^2 \end{aligned}$$

and, hence

$$\begin{aligned} E[S_1S_2]-E[S_1]E[S_2] &= f_{11}-f_{13}-f_{31}+f_{33}-(p_1^2q_1^2-p_2^2q_1^2-p_1^2q_2^2+p_2^2q_2^2) \\ &= (f_{11}-p_1^2q_1^2)-(f_{13}-p_2^2q_1^2)-(f_{31}-p_1^2q_2^2)+(f_{33}-p_2^2q_2^2) \end{aligned}$$

Again, using the relationship in Table 3, we have

$$E[S_1S_2]-E[S_1]E[S_2] = (f_{11}-(x_{11}-D)^2)-(f_{13}-(x_{12}+D)^2)-(f_{31}-(x_{21}+D)^2)+(f_{33}-(x_{22}-D)^2)$$

In conclusion, we have shown that the relationship between LD and correlation between haplotype of two locus is given by

$$\boxed{Cov[ab]=4D}$$

Moreover, there is a relationship between covariance of two SNPs with $\{-1, 0, 1\}$ representation and LD as shown in the following equation.

$$\boxed{Cov[S_1S_2]=(f_{11}-(x_{11}-D)^2)-(f_{13}-(x_{12}+D)^2)-(f_{31}-(x_{21}+D)^2)+(f_{33}-(x_{22}-D)^2)}$$
