# Supplementary material for: iLOCi: a SNP interaction prioritization technique for detecting epistasis in genome-wide association studies
Source: BMC Genomics. 2012 Dec 7;13(Suppl 7):S2. doi: 10.1186/1471-2164-13-S7-S2 (PMC3521387; doi:10.1186/1471-2164-13-S7-S2)
Supplement: Additional file 2 — Penetrance tables for dataset simulation (Penetrance_tables.pdf). This file includes the penetrance models used for dataset simulation of two-locus and high-order ineractions. [file 1471-2164-13-S7-S2-S2.pdf]

Table S1 - Eighteen penetrance models of epistatic interactions without marginal effect

h^2 = 0.4, MAF = 0.2			
model 1	AA	Aa	aa
BB	0.486	0.960	0.538
Bb	0.947	0.004	0.811
Bb	0.640	0.606	0.909
model 2	AA	Aa	aa
BB	0.469	0.956	0.697
Bb	0.945	0.019	0.585
Bb	0.786	0.407	0.013
model 3	AA	Aa	aa
BB	0.498	0.954	0.786
Bb	0.978	0.038	0.428
Bb	0.590	0.821	0.380

h^2 = 0.4, MAF = 0.4			
model 4	AA	Aa	aa
BB	0.077	0.656	0.880
Bb	0.892	0.235	0.312
bb	0.174	0.842	0.106
model 5	AA	Aa	aa
BB	0.895	0.323	0.161
Bb	0.068	0.728	0.806
bb	0.925	0.233	0.362
model 6	AA	Aa	aa
BB	0.805	0.251	0.085
Bb	0.002	0.668	0.638
bb	0.83	0.079	0.542

h^2 = 0.3, MAF = 0.2			
model 7	AA	Aa	aa
BB	0.500	0.926	0.615
Bb	0.895	0.131	0.647
Bb	0.858	0.160	0.999
model 8	AA	Aa	aa
BB	0.413	0.851	0.535
Bb	0.831	0.008	0.580
Bb	0.692	0.268	0.736
model 9	AA	Aa	aa
BB	0.455	0.848	0.897
Bb	0.890	0.088	0.016
Bb	0.562	0.686	0.467

h^2 = 0.3, MAF = 0.4			
model 10	AA	Aa	aa
BB	0.891	0.362	0.480
Bb	0.213	0.829	0.601
bb	0.925	0.267	0.685
model 11	AA	Aa	aa
BB	0.077	0.689	0.417
Bb	0.763	0.150	0.491
bb	0.196	0.657	0.247
model 12	AA	Aa	aa
BB	0.091	0.827	0.863
Bb	0.869	0.393	0.415
bb	0.738	0.508	0.363

h^2 = 0.2, MAF = 0.2			
model 13	AA	Aa	aa
BB	0.428	0.757	0.812
Bb	0.788	0.132	0.044
Bb	0.559	0.548	0.373
model 14	AA	Aa	aa
BB	0.507	0.842	0.605
Bb	0.845	0.162	0.629
Bb	0.581	0.678	0.729
model 15	AA	Aa	aa
BB	0.577	0.247	0.428
Bb	0.227	0.928	0.578
Bb	0.586	0.262	0.158

h^2 = 0.2, MAF = 0.4			
model 16	AA	Aa	aa
BB	0.356	0.891	0.809
Bb	0.955	0.508	0.611
bb	0.617	0.755	0.630
model 17	AA	Aa	aa
BB	0.085	0.339	0.772
Bb	0.513	0.651	0.607
bb	0.250	0.999	0.154
model 18	AA	Aa	aa
BB	0.393	0.764	0.664
Bb	0.850	0.398	0.733
bb	0.406	0.927	0.147

Table S2 - Penetrance models of epistatic interactions with marginal effect

(Moore et al. 2002)

model 1	AA	Aa	aa
BB	0.083	0.076	0.964
Bb	0.056	0.508	0.085
Bb	0.977	0.098	0.062
**model 2**	**AA**	**Aa**	**aa**
BB	0.094	0.905	0.097
Bb	0.967	0.097	0.937
Bb	0.027	0.990	0.080
**model 3**	**AA**	**Aa**	**aa**
BB	0.967	0.314	0.137
Bb	0.313	0.312	0.742
Bb	0.129	0.779	0.075

model 4	AA	Aa	aa
BB	0.967	0.139	0.799
Bb	0.057	0.655	0.627
Bb	0.974	0.544	0.019
**model 5**	**AA**	**Aa**	**aa**
BB	0.017	0.451	0.711
Bb	0.520	0.571	0.039
Bb	0.640	0.053	0.949
**model 6**	**AA**	**Aa**	**aa**
BB	0.954	0.256	0.360
Bb	0.010	0.731	0.300
Bb	0.801	0.093	0.808

Table S3 - Penetrance models of two-locus interactions from Neuman and Rice 1992 used for generation of high-order interaction models

Ep1	AA	Aa	aa
BB	0.707	0.707	0.000
Bb	0.707	0.707	0.000
Bb	0.000	0.000	0.000
Ep3	AA	Aa	aa
BB	0.900	0.000	0.000
Bb	0.000	0.000	0.000
Bb	0.000	0.000	0.000
Ep5	AA	Aa	aa
BB	0.799	0.799	0.000
Bb	0.799	0.000	0.000
Bb	0.000	0.000	0.000
Ep6	AA	Aa	aa
BB	0.000	1.000	1.000
Bb	1.000	0.000	0.000
Bb	1.000	0.000	0.000

Het1	AA	Aa	aa
BB	0.745	0.745	0.495
Bb	0.745	0.745	0.495
bb	0.495	0.495	0.000
Het3	AA	Aa	aa
BB	1.000	1.000	1.000
Bb	1.000	0.000	0.000
bb	1.000	0.000	0.000
S1	AA	Aa	aa
BB	0.522	0.522	0.522
Bb	0.522	0.522	0.522
bb	0.522	0.522	0.000
S3	AA	Aa	aa
BB	1.000	1.000	0.512
Bb	1.000	0.512	0.000
bb	0.512	0.000	0.000
